# Hives in autonomic disorders: a cutaneous marker of a distinct symptom phenotype

**DOI:** 10.1080/07853890.2026.2626224

**Published:** 2026-02-10

**Authors:** Chatuthanai Savigamin, Tae Chung, Alison W. Rebman, Yanni Larsen, Elizabeth Clark, Erica Cerquetti, Christina Kokorelis, Pegah Dehghan, Peter C. Rowe, Brittany L. Adler

**Affiliations:** ^a^ Division of Rheumatology, Johns Hopkins University, Baltimore, MD, USA; ^b^ Department of Physical Medicine and Rehabilitation, Johns Hopkins School of Medicine, Baltimore, MD, USA; ^c^ Department of Molecular & Cell Biology, Johns Hopkins University, Baltimore, MD, USA; ^d^ Division of Pediatric Rehabilitation Medicine, Kennedy Krieger Institute, Baltimore, MD, USA; ^e^ Division of Adolescent and Young Adult Medicine, Department of Pediatrics, Johns Hopkins University School of Medicine, Baltimore, MD, USA

**Keywords:** Postural orthostatic tachycardia Syndrome, hives, urticaria, orthostatic intolerance, mast cell activation syndrome

## Abstract

**Background:**

Postural Orthostatic Tachycardia Syndrome (POTS) and Neurally-Mediated Hypotension (NMH) are heterogeneous syndromes characterized by dysautonomia and multisystem symptoms. Mast cell activation, often manifesting as hives, has been proposed as a contributing mechanism, but its prevalence and clinical relevance in POTS and NMH are poorly defined.

**Method:**

Patients from the Johns Hopkins POTS Clinic completed surveys assessing hives frequency and symptom burden using the Malmö POTS, the Composite Autonomic Symptom Score (COMPASS)-31, and a pain questionnaire. Associations between hives and clinical features were evaluated among patients with confirmed POTS, NMH, or clinically diagnosed orthostatic intolerance.

**Result:**

Among 188 respondents, 80 (42.6%) reported hives sometimes and 33 (17.6%) reported hives often or always. Increasing hives frequency was associated with higher Malmö POTS scores and greater autonomic symptom burden across multiple COMPASS-31 subdomains, including gastrointestinal, bladder, and vasomotor symptoms (all *p* < 0.05). Hives was also associated with pain (OR 3.47, 95% CI 1.54–7.77, *p* = 0.002) and tingling (OR 5.73, CI 2.15–15.26, *p* < 0.001), but not orthostatic symptoms. These associations persisted after multivariable adjustment.

**Conclusion:**

Hives are common in orthostatic intolerance syndromes and are associated with increased symptom burden. Future studies are needed to clarify the role of mast cell activation and evaluate mast cell-targeted therapies.

## Introduction

Orthostatic intolerance, a syndrome that often results from autonomic dysfunction, is rising in prevalence and recognition since the COVID-19 pandemic [1]. The two most common autonomic disorders associated with orthostatic intolerance are Postural Orthostatic Tachycardia Syndrome (POTS) and Neurally-Mediated Hypotension (NMH), which result from dysregulated blood flow upon standing. Symptoms include positional dizziness, palpitations, exertional intolerance, cognitive dysfunction, and severe fatigue [2]. POTS is defined by an excessive increase in heart rate (HR) of more than 30 beats per minute (bpm) in adults upon standing, whereas NMH is characterized by a sudden and delayed drop in systolic blood pressure (BP) ≥25 mmHg from upright posture [3,4]. In addition to these hemodynamic disturbances, patients frequently experience a broad spectrum of non-cardiovascular manifestations, including gastrointestinal (GI) symptoms, urinary dysfunction, sicca symptoms, and chronic pain [5].

A growing body of literature suggests that a subset of patients with POTS also have mast cell activation syndrome (MCAS), a disorder of dysregulated mast cells with excessive mast cell mediator release, which can mimic or exacerbate dysautonomia through symptoms including tachycardia, flushing, GI sensitivities, headache, and cognitive dysfunction. MCAS can also cause more specific disease manifestations such as urticaria or angioedema [6–8]. Up to 42% of patients with POTS have biochemical evidence of mast cell degranulation, marked by elevated histamine or prostaglandins [6]. The autonomic nervous system (ANS) and mast cells are closely interconnected, with the ANS directly modulating mast cell activity through the release of neuropeptides, and mast cell mediators promoting neuroinflammation and ANS dysfunction [9]. This bidirectional relationship may underlie the significant symptom overlap between MCAS and dysautonomia, complicating efforts to differentiate between mast cell-related and autonomic-driven manifestations.

Although a MCAS-like phenotype has been widely described in POTS by experienced clinicians and is often reported to respond to mast cell stabilizers, this classification remains poorly defined. The literature supporting this clinical phenotype is sparse and consists predominately of case series and symptom surveys. No studies to our knowledge have examined the relationship between symptom profiles [6,10].

Among symptoms typically associated with MCAS, hives stand out as a hallmark clinical sign that is visible and objective. While hives are reported in up to 25% of patients with POTS [11], it is unclear if these patients represent a biologically distinct subtype of orthostatic intolerance that may have different diagnostic or treatment paradigms. Alternatively, hives could simply be a downstream manifestation of autonomic dysfunction, lacking independent clinical or pathophysiologic significance.

In this study, we sought to characterize the prevalence of hives in a large cohort of patients with POTS and NMH, and to explore whether hives are associated with distinct symptom profiles, autonomic testing parameters, and small fiber nerve density on skin biopsy. We hypothesized that hives may identify a clinically meaningful subset of dysautonomia characterized by features typically attributed to MCAS. Hives were chosen as a potential predictor of a MCAS-driven phenotype of dysautonomia given their specificity for mast cell activation relative to other more non-specific symptoms like fatigue. These findings may help identify a distinct clinical and pathophysiologic phenotype of orthostatic intolerance, with potential implications for improved diagnostic approaches and targeted therapeutics for POTS and NMH.

## Methods

### Study cohort and design

We conducted a cross-sectional study at the Johns Hopkins POTS Clinic, a large tertiary referral clinic for patients with POTS and autonomic dysfunction. All research studies were performed in accordance with the principles stated in the Declaration of Helsinki. Electronic questionnaires were sent to 851 consecutive clinic patients between 07/2024 and 10/2024 to assess the presence and frequency of hives and their overall symptom burden. Written informed consent was obtained from all participants. We analyzed the association between hives and symptom profiles as well as clinical features. Clinical data were abstracted from the electronic medical record, and included comorbid diagnoses (Ehlers Danlos Syndrome or EDS, Inappropriate Sinus Tachycardia or IST, or Mast Cell Activation Syndrome), current medication use related to POTS or MCAS, autonomic testing results, and small nerve fiber density on skin biopsy.

Given that POTS, NMH, and orthostatic intolerance exist on a spectrum of autonomic dysfunction and often overlap, we included all patients with an abnormal tilt table test or 10 min stand test (diagnostic of POTS and/or NMH) and those being actively managed for clinically significant orthostatic intolerance despite a negative tilt table test. This approach reflects real-world clinical practice, where tilt table testing has limited sensitivity due to numerous factors including daily variability in autonomic function and hydration status [12,13]. Furthermore, patients with longer disease duration may be less likely to have a positive tilt table test [14]. By including patients with orthostatic intolerance, we sought to capture a more comprehensive cohort of patients affected by autonomic dysfunction that reflects the complexity of this disorder beyond POTS and NMH alone.

The tilt table or 10 min stand test was considered abnormal if the interpretation was consistent with POTS and/or NMH. POTS was defined as an increase in heart rate ≥30 bpm from supine to standing for patients ≥18 years or ≥40 bpm for patients <18 years, or an increase in heart rate ≥120 bpm with standing. NMH was defined as a sudden drop in systolic blood pressure ≥25 mmHg [4]. Patients with an immediate drop in their blood pressure within the first 3 min of standing were considered to have neurogenic orthostatic hypotension and were excluded from the analysis, as this diagnosis tends to occur in older patients and likely has a distinct mechanism as it is often associated with neurodegenerative diseases [15]. Autonomic testing was done for clinical purposes and was not standardized in this study. Some patients had a 10 min active stand test, while others had a 10 min tilt table test, and others had an extended tilt table test involving elevation at 70 degrees for up to 45 min (stage 1) followed by another period supine and then 15 min upright with an infusion of isoproterenol (stage 2). Therefore, it is possible that some patients with a negative test may show evidence of POTS or NMH with more extensive testing. For patients with a negative tilt table test, a chart review was conducted to confirm the presence of orthostatic intolerance, based on the presence of orthostatic symptoms.

### Patient questionnaires

#### Flushing and hives

Patients were asked whether they experience ‘flushing’ and ‘hives and/or welts’. Responses were recorded on a Likert scale with the options: ‘never’, ‘sometimes’, ‘often’ or ‘always’. The categories ‘often’ and ‘always’ were combined due to the low number that reported ‘always’ having hives.

#### Malmö POTS

The Malmö POTS questionnaire is a 12-item self-assessment tool based on patients’ perception of both cardiac (e.g. palpitations, dizziness, presyncope) and non-cardiac symptoms (e.g. fatigue, concentration difficulties, gastrointestinal disturbances). Patients score each symptom on a scale from 0 (no symptoms) to 10 (very severe symptoms), with a total score ranging from 0 to 120. This tool has been used in POTS and has excellent sensitivity (97%) and specificity (98%) in distinguishing POTS from healthy controls [16].

#### COMPASS-31

The COMPASS-31 is a validated 31-item questionnaire used to quantify autonomic symptoms across six domains: orthostatic intolerance, vasomotor, secretomotor, gastrointestinal, bladder, and pupillomotor symptoms. Each domain is scored based on symptom frequency and severity, producing a total score from 0 to 100 that reflects overall autonomic symptom burden [17].

#### Pain

Patients were asked whether they experience pain, and those who responded ‘yes’ were asked about the quality of their pain. Specifically, they were asked whether their pain feels like burning, squeezing, pressure, electric shocks, or a stabbing sensation. The duration and frequency of painful episodes were recorded and potential triggers were characterized. These questions were adapted from the core domains of the Neuropathic Pain Symptom Inventory (NPSI) [18]. Because our primary objective was to subgroup patients based on the presence or absence of specific pain phenotypes, the responses were dichotomized (yes/no) to facilitate clinically meaningful clustering and reduce variability introduced by continuous rating scales. All patients were also asked whether they experience pins and needles or tingling, and all responses were recorded as categorical variables.

### Autonomic testing and cutaneous nerve biopsy

Results from autonomic testing and the cutaneous nerve biopsy were obtained by retrospective chart review for patients who underwent these tests for clinical purposes. Although tilt table results from any hospital were used to determine if patients met criteria for POTS or NMH, only data from tilt table tests done at Johns Hopkins were used to correlate with symptoms. The change in heart rate during the first 10 min of tilt was recorded for all patients regardless of whether they underwent the 10 min tilt or more prolonged 45 min procedure. Valsalva Index and heart rate variability on deep breathing were available for patients who had testing performed in the Johns Hopkins Autonomic Lab. For the cutaneous nerve biopsy, a punch biopsy was obtained using a 3-mm Integra punch tool from three standardized skin biopsy sites: the distal leg (lateral), distal thigh (lateral), and proximal thigh (lateral). The site was anesthetized with 1% lidocaine with epinephrine and all tissue samples were placed into Zamboni fixative. Intraepidermal and sudomotor nerve fiber densities were quantified at the distal leg and proximal thigh. Intraepidermal nerve fiber density was additionally measured at the distal thigh, for a total of 5 distinct measurements per patient.

### Statistical analysis

Descriptive statistics were used to summarize patient characteristics and test results. Hives were treated as a binary (yes/no) variable in analyses examining associations with demographic features, autonomic diagnoses, and the presence and quality of pain, to allow for estimation of odds ratios. For analyses of the association between hives and autonomic symptoms or clinical parameters (COMPASS-31, Malmö POTS, tilt table test results, and skin nerve fiber density), hives were treated as an ordinal variable (never/sometimes/often-always) to evaluate trends in symptom frequency and disease severity. The Kruskal-Wallis test was used to assess for group differences for continuous variables and chi-square or Fisher’s exact test for categorical variables. Dunn’s test was used as a post-hoc test to determine which specific group differences were significant following the Kruskal-Wallis test. A multivariable linear regression was used to test the hypothesis that hives frequency predicts symptom severity after adjusting for covariates. Only covariates that were significant in the univariate analysis (age, sex, race, or autonomic diagnosis) were included in the multivariable model. Significance was defined as *p* < 0.05 for all analyses.

## Results

### Patient population

Electronic questionnaires were sent to 851 consecutive patients in the Johns Hopkins POTS Clinic, and 199/851 (23.4%) responded. Eight were excluded because of a questionable diagnosis of orthostatic intolerance, and 3 were excluded because they met criteria for neurogenic orthostatic hypotension. Therefore, a total of 188 patients met criteria for POTS, NMH, and/or orthostatic intolerance and were included in the final analysis.

Of the patients in the final analysis, 177/188 (94.1%) were female, 170/188 (90.4%) were white, and the median age was 35 years [IQR 27–45]. 136/188 (72.3%) had a tilt table test performed. Of the remainder that did not have a tilt table test, 17/52 (32.7%) had a 10-minute stand test. The tilt table test was performed a mean (SD) of 1.98 (2.25) years before the surveys were completed. The majority of tilts occurred at Johns Hopkins in two different labs: 24/136 (17.6%) had testing done in the Johns Hopkins Autonomic Lab which performs a 10-minute tilt, Valsalva maneuver, and heart rate variability on deep breathing. 54/136 (39.7%) had the extended tilt performed in the Johns Hopkins Cardiology Lab. Of the patients who completed the extended tilt, 25 only completed Stage 1, whereas 29 patients completed both Stage 1 and Stage 2 with isoproterenol challenge. 54/188 (28.7%) had a cutaneous nerve biopsy performed at Johns Hopkins, which was performed on average 2.0 ± 0.2 years before the symptom surveys.

102/188 patients (54.3%) met criteria for POTS on either the tilt table test or 10-minute stand test, and 19/188 (10.1%) met criteria for NMH. 8/188 (4.3%) met criteria for both POTS and NMH. The remainder of patients (*n* = 59) had clinically significant orthostatic intolerance defined as increased symptoms when upright, 40.7% of whom (24/59) had a normal tilt and/or 10-minute stand test. The median Malmö POTS score for the orthostatic intolerance group was 77[IQR 65–88], which was not significantly different from the Malmö POTS Score for the patients with confirmed POTS and/or NMH (Kruskal-Wallis Test *p* = 0.416).

#### Demographic and clinical associations with hives

A total of 113/188 patients (60.1%) reported experiencing hives, with 80/188 (42.6%) reporting ‘sometimes’ having hives, and 33/188 (17.6%) reporting ‘often or always’ having hives. We first examined the association between the presence of hives and basic demographic features and autonomic diagnosis (Table 1). We found that the presence of hives was significantly associated with female sex (odds ratio [OR] 4.34, *p* = 0.028). There was no association between hives and age, race, or autonomic diagnosis (orthostatic intolerance, POTS, or NMH) (*p* > 0.05). Patients with hives were more likely to have a diagnosis of MCAS, EDS, or IST (*p* < 0.05) and were more likely to be prescribed antihistamines or cromolyn (*p* < 0.01). There was no association between hives and other hemodynamic medications commonly used to treat POTS (*p* > 0.05).

**Table 1.** Association between hives, demographic characteristics, and autonomic diagnosis.

Demographics and diagnosis		Presence of hives		*p*-Value	Odds ratio [95% CI] (*p* value)
		Yes (*n* = 113)	No (*n* = 75)		
Age, median [IQR]		34.00 [27.00–44.00]	36.00 [28.00–47.50]	Kruskal–Wallis test *p* = 0.163	NA
Sex	Female	110/113 (97.3%)	67/75 (89.3%)	Fisher’s exact test *p* = 0.028	4.34 [1.00–26.29] (*p* = 0.028)
Race	White	103/113 (91.2%)	67/75 (89.3%)	Chi square *p* = 0.678	1.23 [0.46–3.27] (*p* = 0.801)
Autonomic diagnosis	POTS only	68/113 (60.2%)	34/75 (45.3%)	Fisher’s exact *p* = 0.213	NA
	NMH only	10/113 (8.8%)	9/75 (12.0%)		
	POTS and NMH	5/113 (4.4%)	3/75 (4.0%)		
	Orthostatic intolerance	30/113 (26.5%)	29/75 (38.7%)		
Associated diagnosis					
	Inappropriate sinus tachycardia	19/113 (16.8%)	3/75 (4.0%)	Fischer’s exact *p* < 0.001	4.85 [1.38–17.03] (*p* = 0.01)
	Mast cell activation syndrome	64/113 (56.6%)(	21/75 (28.0%)	Chi square *p* < 0.001	3.36 [1.80–6.28] (*p* < 0.001)
	Hypermobility Ehler Danlos syndrome	56/113 (49.6%)	17/75 (22.7%)	Chi square *p* < 0.001	3.35 [1.74–6.45] (*p* < 0.001)
Medication					
	Beta blockers	50/113 (44.2%)	34/75 (45.3%)	Chi square *p* = 0.883	0.96 [0.53–1.72] (*p* = 1.000)
	Midodrine	52/113 (46.0%)	33/75 (44.0%)	Chi square *p* = 0.785	1.09 [0.60–1.95] (*p* = 0.881)
	Fludrocortisone	25/113 (22.1%)	14/75 (18.7%)	Chi square *p* = 0.567	1.24 [0.60–2.57] (*p* = 0.588)
	Ivabradine	18/113 (15.9%)	6/75 (8.0%)	Chi square *p* = 0.111	2.18 [0.82–5.77] (*p* = 0.124)
	Pyridostigmine	28/113 (24.8%)	13/75 (17.3%)	Chi square *p* = 0.226	1.57 [0.75–3.28] (*p* = 0.280)
	Intravenous fluids	8/113 (7.1%)	1/75 (1.3%)	Fisher’s exact test *p* = 0.089	5.63 [0.69–46.0] (*p* = 0.089)
	Antihistamine	65/113 (57.5%)	23/75 (30.7%)	Chi square *p* < 0.001	3.06 [1.65–5.67] (*p* < 0.001)
	Cromolyn	21/113 (18.6%)	4/75 (5.3%)	Fisher’s exact test *p* = 0.009	4.05 [1.33–12.33] (*p* = 0.009)
	SSRI	25/113 (22.1%)	22/75 (29.3%)	Chi square *p* = 0.264	0.68 [0.35–1.33] (*p* = 0.303)
	SNRI	10/113 (8.8%)	9/75 (12.0%)	Chi square *p* = 0.483	0.71 [0.28–1.85] (*p* = 0.622)
	Tricyclic antidepressant	9/113 (8.0%)	4/75 (5.3%)	Fisher’s exact test *p* = 0.569	1.54 [0.46–5.18](*p* = 0.569)
	Low dose naltrexone	25/113 (22.1%)	11/75 (14.7%)	Chi square *p* = 0.203	1.65 [0.76–3.60] (*p* = 0.257)
	Desmopressin	5/113 (4.4%)	3/75 (4.0%)	Fisher’s exact test *p* = 1.000	1.11 [0.26–4.79] (*p* = 1.000)
	Droxidopa	4/113 (3.5%)	2/75 (2.7%)	Fisher’s exact test *p* = 1.000	1.34 [0.24–7.50] (*p* = 1.000)
	Stimulant	30/113 (26.5%)	12/75 (16.0%)	Chi square *p* = 0.089	1.90[0.90–4.00] (*p* = 0.108)

The Kruskal Wallis test was used to detect group differences for continuous variables. The chi-square test and Fischer exact test were used for categorical variables. Fisher exact test was used if any subgroup had less than 5 participants. *p* < 0.05 was considered significant.

**Figure 1.**
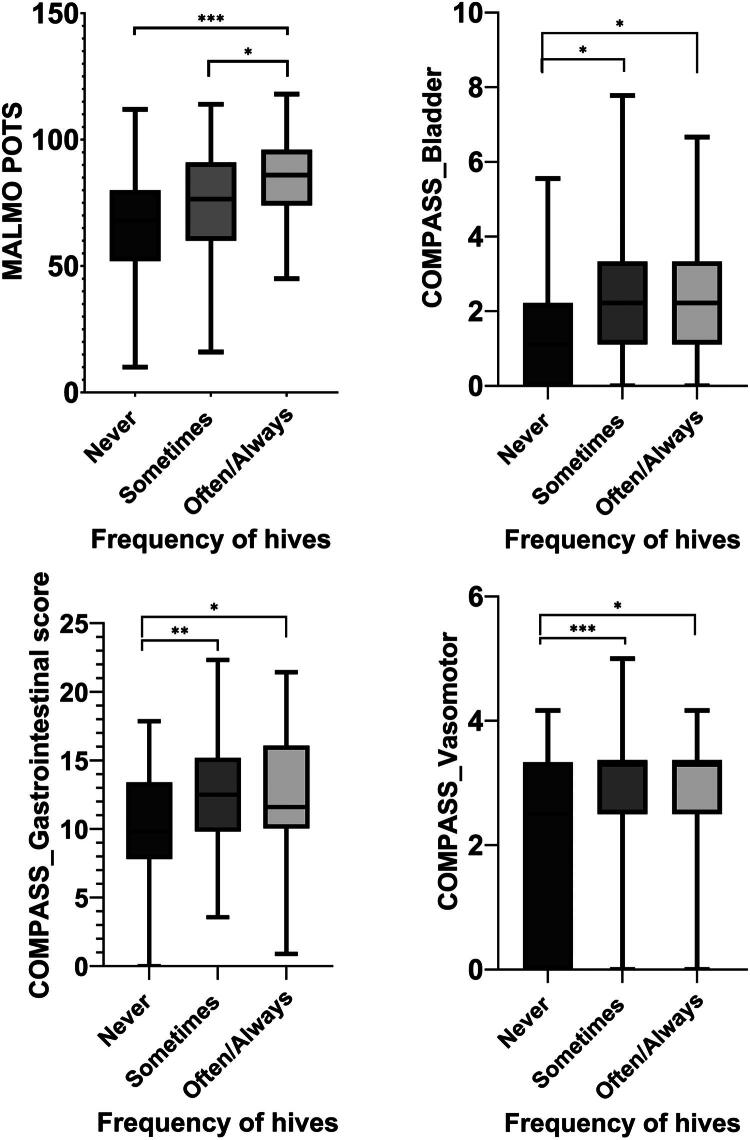
Symptom burden and frequency of hives among patients with postural orthostatic tachycardia syndrome (POTS), neurally-mediated hypotension (NMH), and clinical orthostatic intolerance. Symptoms were measured by the Malmö POTS and COMPASS-31 questionnaires. There was no difference in the orthostatic intolerance, secretomotor, or pupillomotor subdomains by frequency of hives. Median and interquartile range are presented. *p < 0.05, ** *p* < 0.01, *** *p* < 0.001.

We then examined the association between hives and symptom burden as measured by the Malmö POTS and COMPASS-31 questionnaires. For this analysis, hives were treated as an ordinal variable (never/sometimes/often-always) to evaluate trends in symptom severity by the frequency of hives. We identified a significant positive association between the frequency of hives and a higher Malmö POTS score (*p* < 0.001) and COMPASS-31 gastrointestinal (*p* = 0.006), bladder (*p* = 0.013), and vasomotor (*p* = 0.002) subdomain scores (Figure 1, Table S1). The association with the COMPASS-31 GI domain was explained by significant associations between hives and abdominal pain (*p* = 0.004) and diarrhea (*p* = 0.049), but not early satiety, vomiting or constipation (*p* > 0.05). There was no association between hives and secretomotor, pupillomotor, or orthostatic subdomains (*p* > 0.05).

**Figure 2.**
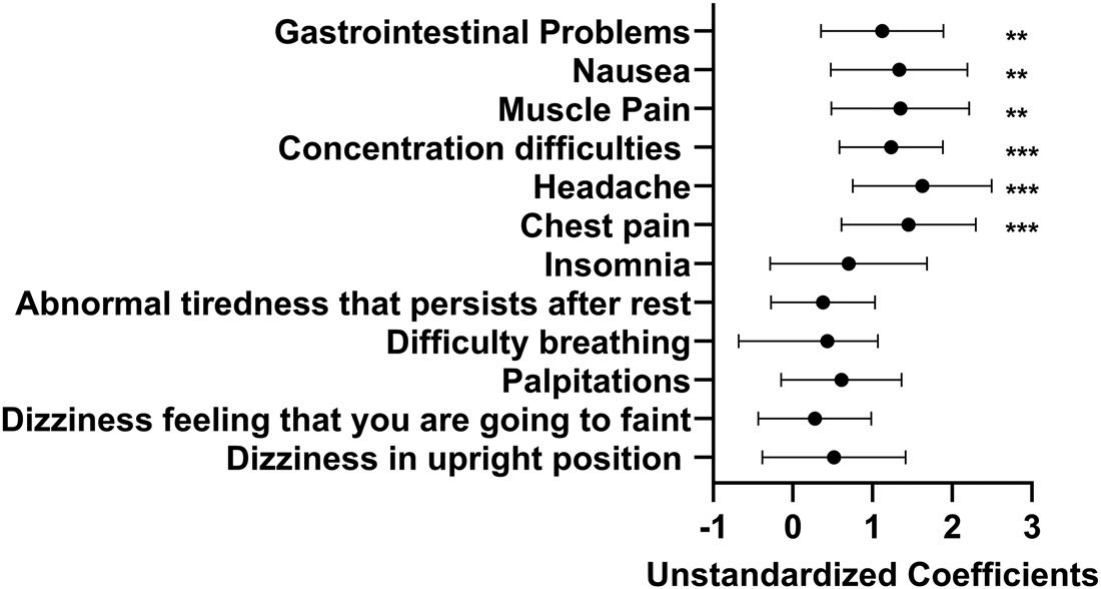
The difference in symptom burden between patients with and without hives among 188 patients with postural orthostatic tachycardia syndrome (POTS), neurally-mediated hypotension (NMH), and/or clinical orthostatic intolerance. Displayed are the coefficient estimate and 95% confidence limits for separate linear regression models with each symptom of the Malmö POTS questionnaire as the outcome and hives as the primary predictor of interest. All 12 of the symptoms tested are displayed. *p < 0.05, **p < 0.01, ***p < 0.001.

Specific symptoms queried in the Malmö POTS questionnaire that were significantly associated with hives included chest pain, headache, concentration difficulty, muscle pain, nausea, GI problems, and palpitations (*p* < 0.05). There was no association with symptoms of dizziness, fainting, shortness of breath, abnormal tiredness, or insomnia (*p* > 0.05) (Table S2). Figure 2 shows the difference in symptom burden between patients with and without hives for each item of the Malmö POTS questionnaire, as determined by separate linear regression models with each symptom of the Malmö POTS questionnaire as the outcome and hives as the predictor. This analysis again showed an association between hives and specific symptom profiles.

We then constructed a multivariable linear regression model to assess whether hives predict symptom severity in patients with POTS, NMH, or orthostatic intolerance after adjusting for potential covariates (age, sex, race, and autonomic diagnosis). The frequency of hives remained a significant predictor of the Malmö POTS and COMPASS-31 GI, bladder, and vasomotor subdomain scores after adjusting for covariates that were significant in the univariate analysis (Table 2, Table S3).

**Table 2.** Multivariable linear regression model assessing the association between hives and clinical symptoms among patients with orthostatic intolerance syndromes.

Dependent variable	Covariate	Adjusted model ± Standard error	*t*	*p* Value
Malmö POTS (*R*^2^= 0.161)	Hives (often/always) Hives (sometimes)	16.35 ± 4.19 6.88 ± 3.17	3.90 2.17	*p* < 0.001 *p* 0.031
	Age	−0.24 ± 0.11	−2.07	*p* = 0.040
	Sex (female)	7.04 ± 6.17	1.14	*p* = 0.255
	Race (White)	−12.47 ± 4.84	−2.58	*p* = 0.011
COMPASS-31 – bladder score (*R*^2^ = 0.051)	Hives (often/always) Hives (sometimes)	0.93 ± 0.35 0.67 ± 0.27	2.69 2.53	*p* = 0.008 *p* = 0.012
COMPASS-31 –gastrointestinal score (*R*^2^ = 0.077)	Hives (often/always) Hives (sometimes)	1.63 ± 0.87 1.79 ± 0.67	1.87 2.66	*p* = 0.009 *p* = 0.046
	Sex (female)	2.86 ± 1.42	2.01	*p* = 0.046
COMPASS-31 –vasomotor Score (*R*^2^= 0.067)	Hives (often/always) Hives (sometimes)	0.63 ± 0.29 0.78 ± 0.22	2.18 3.50	*p* = 0.031 *p* < 0.001
Intraepidermal nerve fiber density at distal leg (*R*^2^= 0.259)	Hives (often/always) Hives (sometimes)	1.97 ± 2.14 −0.53 ± 1.65	0.92 −0.32	*p* = 0.362 *p* = 0.750
	Age	−0.20 ± 0.06	−3.09	*p* = 0.003

Covariates that were included were age, sex, race, and autonomic diagnosis (POTS, NMH, or orthostatic intolerance). Only those that were significant in the univariate analysis were retained in the final model. Residuals were checked and were well aligned. *p < 0.05; **p < 0.01; ***p < 0.001.

We next evaluated the association between hives and objective parameters on autonomic testing and cutaneous nerve biopsy (Table S1). There was no association between the frequency of hives and autonomic parameters on the tilt table test, including the change in heart rate with tilt, heart rate variability on deep breathing, or the Valsalva ratio. We observed significant associations between hives and intraepidermal nerve fiber density at the distal leg, with higher nerve fiber density among patients with hives (*p* = 0.047). However, this association was no longer significant after adjusting for age in a multivariable linear regression model (Table 2, Table S3).

We also examined the relationship between hives and the presence and quality of pain (Table 3, Table S4). Patients with hives were more likely to experience pain (OR 3.5, *p* = 0.002). More specifically, patients with hives reported more burning pain (OR 1.98, *p* = 0.033), pressure pain (OR 1.98, *p* = 0.033), and electric shock pain (OR 2.48, *p* = 0.006), but there was no significant difference in squeezing or stabbing pain (*p* > 0.05). Moreover, patients with hives had a greater duration of pain and more frequent painful episodes over the prior 24 h than patients without hives (*p* < 0.05). Patients with more frequent hives were also more likely to have their pain triggered by pressure or something cold compared to those without hives (*p* < 0.05), but there was no association with brushing as a trigger of pain (*p* > 0.05). Patients with more frequent hives were more likely to report tingling (OR 5.73, *p* < 0.001), but not flushing or pins and needles (*p* > 0.05).

**Table 3.** Association between hives and clinical characteristics.

Clinical features		Presence of hives		*p* value	odds ratio [95% CI] (*p* value)
		Yes (*n* = 113)	No (*n* = 75)		
Pain	Yes	101/112 (90.2%)	53/73 (72.6%)	Chi square *p* = 0.002	3.47 [1.54–7.77] (*p* = 0.002)
Burning pain	Yes	58/112 (51.8%)	25/71 (35.2%)	Chi square *p* = 0.028	1.98 [1.07–3.65] (*p* = 0.033)
Squeezing pain	Yes	44/112 (39.3%)	20/71 (28.1%)	Chi square *p* = 0.124	1.65 [0.87–3.13] (*p* = 0.153)
Pressure pain	Yes	82/112 (73.2%)	38/73 (52.0%)	Chi square *p* = 0.003	2.52 [1.35–4.69] (*p* = 0.004)
Electric shock pain	Yes	56/112 (50.0%)	21/73 (28.8%)	Chi square *p* = 0.004	2.48 [1.32–4.64] (*p* = 0.006)
Stabbing pain	Yes	72/111 (64.9%)	39/72 (54.2%)	Chi square *p* = 0.148	1.56 [0.85–2.86] (0.165)
During the past 24 hrs, your spontaneous pain has been present	Less than 1 hr	21/112 (18.8%)	28/73 (38.4%)	Chi square *p* = 0.022	NA
	1–3 hrs	18/112 (16.1%)	14/73 (19.2%)		
	4–7 hrs	22/112 (19.6%)	8/73 (11.0%)		
	8–12 hrs	24/112 (21.4%)	13/73 (17.8%)		
	Permanently	27/112 (24.1%)	10/73 (13.7%)		
Frequency of pain in the past 24 hrs.	None	22/111 (19.8%)	26/72 36.1%)	Fisher’s exact *p* = −0.052, *p* = 0.049	NA
	1 to 5	53/111 (47.7%)	34/72 (47.2%)		
	6 to 10	19/111 (17.1%)	6/72 (8.3%)		
	11 to 20	10/111 (9.0%)	2/72 (2.8%)		
	More than 20	7/111 (6.3%)	4/72 (5.6%)		
Pain provoked by brushing on the painful area	Yes	31/112 (27.7%)	17/72 (23.6%)	Chi Square *p* = 0.540	1.24 [0.63–2.45] (*p* = 0.608)
Pain provoked by pressure on the painful area	Yes	62/111 (55.9%)	29/72 (40.3%)	Chi square *p* = 0.039	1.88 [1.03–3.43] (*p* = 0.049)
Provoked by contact with something cold on the painful area	Yes	34/111 (30.6%)	12/70 (17.1%)	Chi square *p* = 0.042	2.13 [1.02–4.48] (*p* = 0.054)
Pins and needles	Yes	93/113 (82.3%)	53/74 (71.6%)	Chi square *p* = 0.084	1.84 [0.92–3.71] (*p* = 0.104)
Tingling	Yes	107/113 (94.7%)	56/74 (75.7%)	Chi-square *p*< 0.001	5.73 [2.15–15.26] (*p* < 0.001)
Flushing	Yes	106/113 (93.8%)	65/74 (87.8%)	Chi-square *p*= 0.154	2.10 [0.75–5.90] (*p* = 0.185)

Chi-square test and Fischer exact test were used for categorical variables. Fisher exact test will be used if any subgroup has less than 5 participants. Odds ratios were analyzed with 95% confidential interval. *p* < 0.05 was considered significant.

Lastly, to confirm that our findings are robust in a population with objective evidence of POTS and/or NMH, we conducted a sensitivity analysis for Malmö-POTS, COMPASS-31, and pain limited to patients with an abnormal tilt table test (*n* = 129). In this cohort, hives remained significantly associated with higher Malmö POTS scores (Often/Always > Never, *p* < 0.001; Often/Always > Sometimes, *p* = 0.010), as well as increased COMPASS-31 vasomotor (Sometimes > Never, *p* < 0.001) and urinary (Sometimes > Never, *p* = 0.011) symptom scores, with a trend toward significance for GI symptoms (Sometimes > Never, *p* = 0.054). We also observed an association between hives and pain (OR 1.76 [0.71–2.80], *p* < 0.001) and tingling (OR 2.08 [0.89–3.28], *p* < 0.001).

We did not observe a significant association between the presence of flushing and COMPASS-31 or Malmö POTS scores. However, flushing was significantly associated with several pain-related symptoms, including increased overall pain (OR 4.67 [1.59–13.72], *p* = 0.008), increased pressure-evoked pain (OR 3.42 [1.18–9.91], *p* = 0.017), and increased electric-shock–like pain (OR 5.51 [1.21–25.01], *p* = 0.016). These associations persist after adjusting for age, sex, race, and autonomic diagnosis. No significant associations were identified between flushing and autonomic testing parameters. Similar to the findings for hives, we initially observed a significant association between flushing and small nerve fiber density at the proximal thigh (*p* = 0.041); however, this association was no longer significant after adjustment for age (*p* = 0.279).

## Discussion

**Figure 3.**
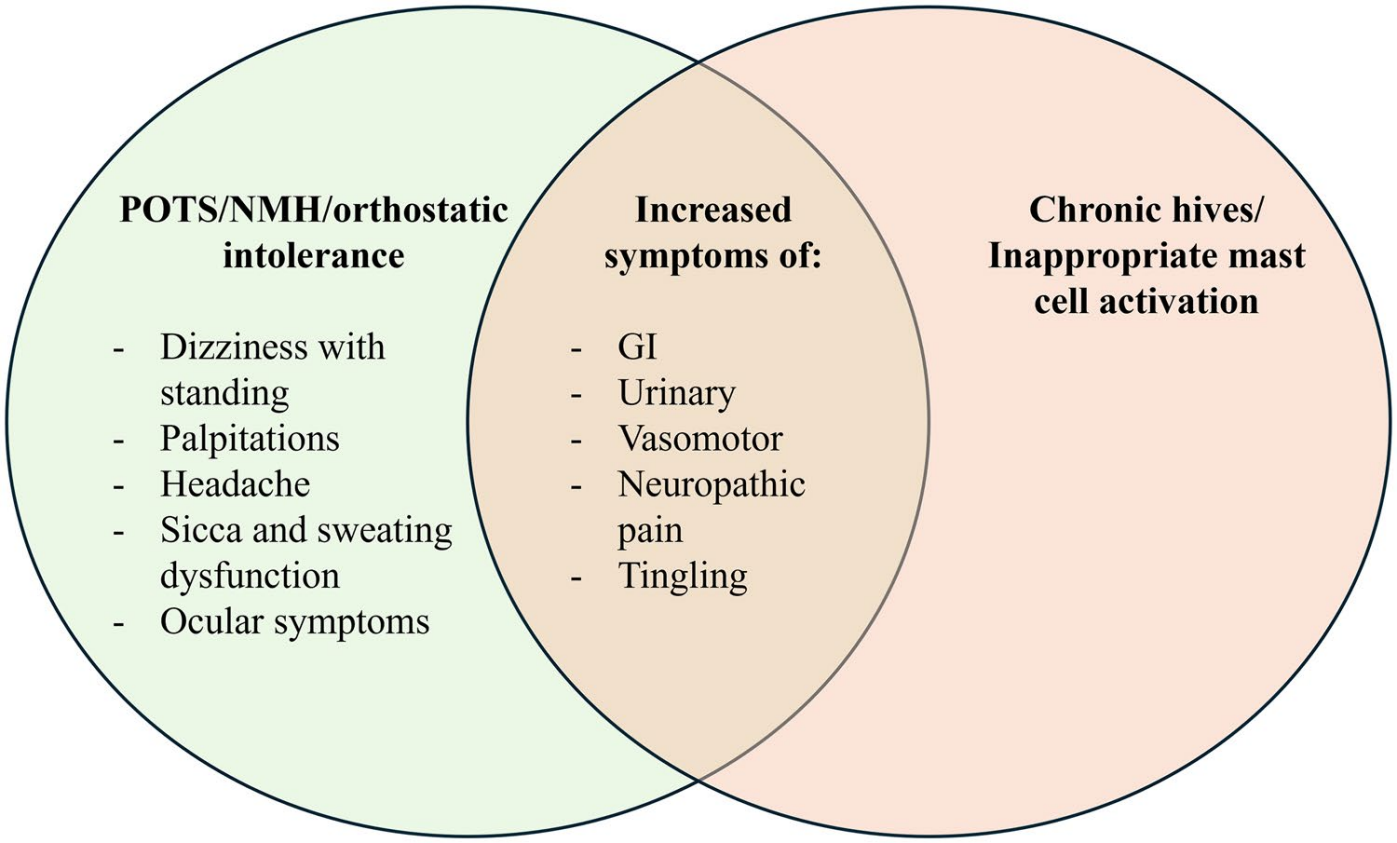
Symptom overlap between orthostatic intolerance syndromes and chronic hives/mast cell activation. Hives are common in Postural Orthostatic Tachycardia Syndrome (POTS) and Neurally Mediated Hypotension (NMH), and are linked to greater symptom burden, especially gastrointestinal (GI), urinary, and vasomotor symptoms, as well as neuropathic pain.

We demonstrate that hives are a common feature of POTS and NMH, occurring in over 60% of patients, and the presence and frequency of hives are associated with more severe GI, urinary, and vasomotor symptoms, as well as increased neuropathic pain. A graphical abstract of these findings is presented in Figure 3. While hives have previously been reported as a common symptom in patients with POTS/NMH in case series [11], to our knowledge this is the first quantitative analysis of hives in a diverse cohort of patients with POTS and NMH.

POTS and NMH are poorly understood syndromes, and the mechanisms underlying autonomic dysfunction in these conditions remain unknown and are likely heterogeneous. Our finding that a large subset of patients with POTS and NMH experience frequent hives, which is typically a manifestation of mast cell activation, provides preliminary evidence that inappropriate mast cell activation may contribute to these syndromes in some cases. Further research is needed to clarify the mechanisms linking autonomic dysfunction and mast cell activation. Given the close interaction between the autonomic nervous system and mast cells, it is possible that autonomic dysfunction leads to an elevated sympathetic state that in turn triggers mast cell degranulation [19]. Conversely, mast cells may directly contribute to neuroinflammation and autonomic dysfunction [20]. Mast cells have been shown to play an important role in vascular regulation by releasing vasoactive mediators such as histamine, prostaglandins, and bradykinin, which could underlie the vasomotor symptoms observed [21] These mediators are also known to enhance nociceptive signaling and promote pain hypersensitivity [9], potentially explaining why patients with hives reported greater pain. Similarly, the high density of mast cells in tissues that modulate smooth muscle function [21] may provide a mechanistic link between hives and urinary and GI symptoms in POTS and NMH.

Our study examined the relationship between symptoms suggestive of mast cell activation and autonomic syndromes, specifically POTS and NMH. However, the clinical overlap between mast cell activation and autonomic dysfunction may extend beyond these specific syndromes. Patients with MCAS frequently experience symptoms consistent with autonomic dysfunction such as orthostatic intolerance and GI symptoms, and studies have identified altered autonomic responses in patients with MCAS [8]. This raises the possibility that a subset of patients across these diagnoses may share a common underlying pathophysiology and represent interrelated conditions rather than distinct disease entities. Further studies incorporating objective autonomic testing and immunologic profiling will be important to delineate shared and distinct mechanisms across these syndromes. Clarifying whether they represent distinct disorders with overlapping symptoms or lie along a disease spectrum will be essential for developing more precise diagnostic tools and targeted therapeutic strategies.

Notably, many of the symptoms associated with hives identified by the COMPASS-31 and Malmö POTS questionnaire were not strictly orthostatic in nature. This observation highlights that a substantial component of symptom burden in this population may arise from non-orthostatic mechanisms. Consequently, therapeutic strategies that target hives or other allergic manifestations such as antihistamines or mast cell–directed treatments may help alleviate broader, non-orthostatic symptoms and improve overall patient-reported outcomes beyond conventional orthostatic management alone.

A major strength of this study is its large cohort of POTS and NMH patients, many of whom had diagnoses confirmed via tilt table testing. However, because tilt table tests were conducted for clinical purposes, the protocols were not standardized; some patients underwent prolonged testing, while others only had a 10-minute tilt. As a result, it is possible that some patients with POTS may have been diagnosed with NMH if they had undergone more prolonged testing. To account for this diagnostic uncertainty, we analyzed all patients together who had the clinical syndrome. Future studies using standardized tilt protocols are needed to clarify potential differences between POTS and NMH. We also lacked a non-POTS control population, which limits our ability to conclude that hives, flushing, and pain are more common in POTS compared to the general population. Another limitation is that hives were self-reported rather than physician-confirmed, and the low response rate may have introduced sample bias. Future studies should better characterize hives in these patients, exploring potential triggers (e.g. heat, stress) and the temporal relationship between hives onset and POTS/NMH symptoms.

Our study is the first to provide strong evidence that hives are associated with a distinct symptom profile in POTS and NMH, suggesting they may define a novel clinical subset. Future research is needed to determine whether this subset has an unique pathophysiology and whether targeted mast cell-directed therapies may be effective.

## Supplementary Material

Supplementary Table.docx

## Data Availability

The datasets used and/or analysed during the current study are available from the corresponding author on reasonable request.
