# Supplementary material for: Hives in autonomic disorders: a cutaneous marker of a distinct symptom phenotype
Source: Ann Med. 2026 Feb 10;58(1):2626224. doi: 10.1080/07853890.2026.2626224 (PMC12895873; doi:10.1080/07853890.2026.2626224)
Supplement: Supplementary Table.docx [file IANN_A_2626224_SM7941.docx]

**Supplementary Table 1. Association between the frequency of hives, symptom burden, and objective measures of autonomic dysfunction and cutaneous small fiber nerve density.** Symptom burden was measured by the Malmö POTS and COMPASS-31 total and subdomain scores.

|  | | **Frequency of hives (Median, IQR) (n)** | | | **Kruskal -Wallis Test (Dunn post-hoc)** |
| --- | --- | --- | --- | --- | --- |
|  |  | **Never**  **(N=75)** | **Sometimes (N=80)** | **Often/Always**  **(N=33)** |  |
| Malmö POTS |  | 68.00,  52.00 – 79.50 (n=75) | 76.50,  60.00 –91.00  (n =80) | 86,  74.00 – 94.00  (n=33) | Overall p < 0.001  Never-Often/Always p < 0.001  Never-Sometimes p = 0.042  Often/Always - Sometimes p = 0.042 |
| COMPASS-31 | Total score | 46.40,  40.36 – 54.31  (n=74) | 51.99,  40.79 – 59.58  (n=78) | 52.01,  42.26 – 59.97  (n=32) | Overall p = 0.242  Never-Often/Always p = 0.180  Never-Sometimes p = 0.142  Often/Always- Sometime p = 0.829 |
|  | Bladder score | 1.11,  0.00 – 2.22  (n=74) | 2.22,  1.11 – 3.33  (n=78) | 2.22,  1.11 – 3.33  (n=32) | Overall p = 0.013  Never-Often/Always p = 0.011  Never-Sometimes p = 0.016  Often/Always- Sometimes p= 0.487 |
|  | Gastrointestinal score | 9.82,  8.04 – 13.39  (n=74) | 12.50,  9.82 – 15.18  (n=78) | 11.61,  10.49 – 16.07  (n=32) | Overall p = 0.006  Never-Often/Always p = 0.025  Never-Sometimes p = 0.003  Often/Always- Sometimes p = 0.950 |
|  | Orthostatic score | 26.00,  20.00 – 32.00  (n=74) | 24.00,  20.00 – 24.00  (n=78) | 24,  20.00 – 24.00  (n=32) | Overall p = 0.913  Never-Often/Always p = 0.805  Never-Sometimes p = 0.039  Often/Always- Sometimes p = 0.940 |
|  | Pupil motor score | 2.66,  2.00 – 3.33  (n=74) | 3.16,  2.41 – 3.66  (n=78) | 3.33,  1.92 – 3.66  (n=32) | Overall p = 0.126  Never-Often/Always p = 0.185  Never-Sometimes p = 0.052  Often/Always- Sometimes p = 0.868 |
|  | Secretory score | 6.43,  4.29 – 8.57  (n=74) | 6.43,  2.14 – 8.57  (n=78) | 6.43,  4.29 – 8.57  (n=32) | Overall p = 0.602  Never-Often/Always p = 0.978  Never-Sometimes p = 0.349  Often/Always- Sometimes p = 0.486 |
|  | Vasomotor Score | 2.50,  0.00 – 3.33  (n=74) | 3.33,  2.50 – 3.33  (n=78) | 3.33,  2.50 – 3.33  (n=32) | Overall p = 0.002  Never-Often/Always p = 0.036  Never-Sometimes p < 0.001  Often/Always- Sometimes p = 0.621 |
| Tilt table parameter | Change in HR during first 10 minutes of tilt | 31.30,  20.00 – 48.00  (n =31) | 35.89,  26.50 – 47.75  (n=34) | 29.00,  21.97 – 37.69  (n=11) | Overall p = 0.452  Never-Often/Always p = 0.583  Never-Sometimes p = 0.396  Often/Always- Sometimes p = 0.244 |
|  | Heart rate variability | 17.05,  15.34 – 25.10  (n=9) | 24.26,  15.30 – 27.10  (n=9) | 22.94,  19.95 – 25.08  (n=5) | Overall p = 0.881  Never-Often/Always p = 0.991  Never-Sometimes p = 0.651  Often/Always- Sometimes p = 0.694 |
|  | Valsalva Index Rate Ratio | 2.12,  1.76 – 2.23  (n=10) | 2.03,  1.83 – 2.29  (n=9) | 2.44,  1.79 – 2.46  (n=5) | Overall p = 0.671  Never-Often/Always p = 0.394  Never-Sometimes p = 0.559  Often/Always- Sometimes p = 0.723 |
| Intraepidermal nerve fiber density | Distal leg | 5.70,  5.05 – 7.93  (n=14) | 6.05,  4.53 – 11.08  (n=26) | 9.90,  8.30 – 16.55  (n=11) | Overall p = 0.047  Never-Often/Always p = 0.021  Never-Sometimes p = 0.641  Often/Always- Sometimes p = 0.032 |
|  | Proximal thigh | 12.55,  8.90 – 16.65  (n=14) | 11.60,  8.08 – 15.35  (n=26) | 11.30,  9.70 – 16.60  (n+11) | Overall p = 0.941  Never-Often/Always p = 0.876  Never-Sometimes p = 0.729  Often/Always- Sometimes p = 0.885 |
| Sudomotor nerve fiber density | Distal leg | 10.20,  8.70 – 11.80  (n=9) | 10.20,  8.90 – 12.90  (n=17) | 8.80,  6.20 – 10.40  (n=9) | Overall p = 0.439  Never-Often/Always p = 0.245  Never-Sometimes p = 0.828  Often/Always- Sometimes p = 0.266 |
|  | Distal Thigh | 15.20,  13.55 – 17.80  (n=7) | 12.70,  9.90 – 15.10  (n=19) | 13.00,  11.10 – 14.25  (n=7) | Overall p = 0.218  Never-Often/Always p = 0.146  Never-Sometimes p = 0.099  Often/Always- Sometimes p = 0.914 |
|  | Proximal thigh | 13.20,  12.48 – 15.65  (n =6) | 14.10,  13.70 – 14.10  (n=5) | 11.00,  9.40 – 12.00  (n=5) | Overall p = 0.140  Never-Often/Always p = 0.083  Never-Sometimes p = 0.945  Often/Always- Sometimes p = 0.084 |

*.*

**Supplementary Table 2. Association between the frequency of hives and symptom burden for each item of the Malmö POTS questionnaire.** The Kruskal-Wallis test was used for overall comparisons, followed by Dunn’s post hoc test for pairwise analyses.

| **Malmö POTS questions** | **Frequency of hives** |
| --- | --- |
| Dizziness in upright position or while standing up | Overall p = 0.077  Never-Often/Always p = 0.048  Never-Sometimes p = 0.812  Often/Always - Sometimes p = 0.029 |
| Dizziness, feeling that you are going to faint | Overall p = 0.110  Never-Often/Always p = 0.042  Never-Sometimes p = 0.731  Often/Always - Sometimes p = 0.073 |
| Palpitations, high pulse, or feeling heart beating irregularly. | Overall p = 0.046  Never-Often/Always p = 0.015  Never-Sometimes p = 0.572  Often/Always - Sometimes p = 0.043 |
| Difficulty breathing/dyspnea, both at effort and rest | Overall p = 0.099  Never-Often/Always p = 0.076  Never-Sometimes p = 0.678  Often/Always - Sometimes p = 0.035 |
| Chest pain | Overall p < 0.001  Never-Often/Always p < 0.001  Never-Sometimes p = 0.012  Often/Always - Sometimes p = 0.099 |
| Headache | Overall p < 0.001  Never-Often/Always p < 0.001  Never-Sometimes p = 0.009  Often/Always - Sometimes p = 0.105 |
| Concentration difficulties and/or problems with thinking | Overall p < 0.001  Never-Often/Always p < 0.001  Never-Sometimes p = 0.006  Often/Always - Sometimes p = 0.105 |
| Muscle pain | Overall p = 0.002  Never-Often/Always p < 0.001  Never-Sometimes p = 0.031  Often/Always - Sometimes p = 0.090 |
| Nausea | Overall p = 0.003  Never-Often/Always p = 0.001  Never-Sometimes p = 0.023  Often/Always - Sometimes p = 0.150 |
| Gastrointestinal Problems (stomach-ache, diarrhea, constipation) | Overall p = 0.035  Never-Often/Always p = 0.013  Never-Sometimes p = 0.093  Often/Always - Sometimes p = 0.231 |
| Abnormal tiredness that persists after rest | Overall p = 0.407  Never-Often/Always p = 0.270  Never-Sometimes p = 0.256  Often/Always - Sometimes p = 0.817 |
| Insomnia | Overall p = 0.086  Never-Often/Always p = 0.027  Never-Sometimes p = 0.461  Often/Always - Sometimes p = 0.097 |

**Supplementary Table 3.** Univariate linear regression analyses assessing the association between potential covariates and symptom/disease burden. Symptom burden was measured by the Malmö POTS and COMPASS-31 total and subdomain scores. Disease burden was measured by autonomic testing parameters and cutaneous small fiber nerve density. Analyses were limited to variables that demonstrated a significant association in preliminary bivariate tests (Chi-square or Kruskal-Wallis). Covariates included hives, age, sex, race, and autonomic diagnosis (POTS, NMH, or orthostatic intolerance). Variables found to be significant in the analysis were considered for inclusion in the multivariable model.

| **Dependent Variable** | **Potential covaiates** | **Regression coefficient (β)± Standard error** | **t-statistic** | **Justification for Inclusion (Statistical / Theoretical)** | **Include in final model** |
| --- | --- | --- | --- | --- | --- |
| Malmö POTS | Age | -0.349 ± 0.117 | -2.99 | p = 0.003 | Yes |
|  | Sex | -12.75 ± 6.46 | -1.97 | p = 0.050 | Yes |
|  | Race | 12.64 ± 5.12 | 2.468 | p = 0.014 | Yes |
|  | Autonomic diagnosis | 7.83 ± 5.23  (POTS only - NMH only )  7.68 ± 8.83  (POTS and NMH- NMH only )  9.36 ± 5.53  (Orthostatic Intolerance - NMH only) | 1.50  0.87  1.69 | p = 0.136  p = 0.390  p = 0.092 | No |
|  | Hive (Often/Always) | 18.65 ± 4.18 | 4.47 | p < 0.001 |  |
|  | Hive (Sometimes) | 7.51 ± 3.21 | 2.34 | p = 0.020 |  |
| COMPASS-31 - Bladder score | Age | 0.003 ± 0.01 | 0.31 | p = 0.757 | No |
|  | Sex | -0.25 ± 0.57 | -0.44 | p = 0.663 | No |
|  | Race | -0.26 ± 0.41 | -0.66 | p = 0.527 | No |
|  | Autonomic diagnosis | 0.43 ± 0.42  (POTS only - NMH only )  0.43 ± 0.73  (POTS and NMH- NMH only )  -0.14 ± 0.44  (Orthostatic Intolerance - NMH only) | 1.04  0.58  -0.33 | p = 0.302  p = 0.562  p = 0.744 | No |
|  | Hive (Often/Always) | 0.93 ± 0.35 | 2.69 | p = 0.008 |  |
|  | Hive (Sometimes) | 0.67 ± 0.27 | 2.53 | p = 0.012 |  |
| COMPASS-31 -Gastrointestinal Score | Age | -0.05 ± 0.02 | -1.91 | p = 0.058 | No |
|  | Sex | -3.74 ± 1.41 | -2.66 | p = 0.009 | Yes |
|  | Race | 0.89 ± 1.04 | 0.86 | p = 0.391 | No |
|  | Autonomic diagnosis | -0.15 ± 1.05  (POTS only - NMH only )  2.10 ± 1.85  (POTS and NMH- NMH only )  -0.38 ± 1.11  (Orthostatic Intolerance - NMH only) | -0.14  1.13  -0.34 | p = 0.885  p = 0.259  p = 0.731 | No |
|  | Hive (Often/Always) | 1.94 ± 0.86 | 2.24 | p = 0.026 |  |
|  | Hive (Sometimes) | 2.06 ± 0.66 | 3.10 | p = 0.002 |  |
| COMPASS-31 -Vasomotor | Age | -0.01 ± 0.01 | -1.33 | p = 0.185 | No |
|  | Sex | -0.38 ± 0.48 | -0.78 | p = 0.434 | No |
|  | Race | -0.20 ± 0.35 | -0.56 | p = 0.580 | No |
|  | Autonomic diagnosis | 0.52 ± 0.36  (POTS only - NMH only )  0.16 ± 0.63  (POTS and NMH- NMH only )  0.31 ± 0.37  (Orthostatic Intolerance - NMH only) | 1.47  0.25  0.82 | p = 0.144  p = 0.803  p = 0.411 | No |
|  | Hive (Often/Always) | 0.63 ± 0.29 | 2.18 | p = 0.031 | Yes |
|  | Hive (Sometimes) | 0.78 ± 0.22 | 3.50 | p < 0.001 | Yes |
| Intraepidermal nerve fiber density at distal leg | Age | -0.22 ± 0.06 | -3.81 | p < 0.001 | Yes |
|  | Sex | -3.89 ± 3.21 | -1.21 | p = 0.231 | No |
|  | Race | -1.415 ± 3.25 | -0.46 | p = 0.666 | No |
|  | Autonomic diagnosis | 1.80 ± 2.06  (POTS only - NMH only )  -0.16 ± 3.65  (POTS and NMH- NMH only )  1.40 ± 2.37  (Orthostatic Intolerance - NMH only) | 0.87  -0.05  0.59 | p = 0.388  p = 0.964  p = 0.559 | No |
|  | Hive (Often/Always) | 4.74 ± 2.10 | 2.25 | p = 0.029 |  |
|  | Hive (Sometimes) | 0.77 ± 1.73 | 0.44 | p = 0.659 |  |

**Supplementary Table 4** Univariate logistic regression analyses assessing the association between potential covariates and the presence of clinical pain. Analyses were limited to variables that demonstrated a significant association with pain in preliminary bivariate tests (Chi-square or Kruskal-Wallis). Covariates included hives, age, sex, race, and autonomic diagnosis (POTS, NMH, or orthostatic intolerance). Variables found to be significant in this analysis were considered for inclusion in the multivariable model.

| **Dependent variable** | **Covariate** | **Unadjusted model ± Standard error** | **z** | **Wald Test**  **p value** | **Include in final model** |
| --- | --- | --- | --- | --- | --- |
| Pain | Hive (Yes) | 1.24 ± 0.41 | 3.02 | 0.003 | Yes |
|  | Age | 0.00 ± 0.015 | 0.01 | 0.992 | No |
|  | Sex (Female) | 0.11 ± 0.81 | 0.13 | 0.896 | No |
|  | Race (White) | 0.74 ± 0.57 | 1.23 | 0.195 | No |
|  | Autonomic diagnosis | -0.32 ± 0.80  (POTS only - NMH only )  -1.57 ± 1.05  (POTS and NMH- NMH only )  -0.65 ± 0.82  (Orthostatic Intolerance - NMH only) | -0.40  -1.50  -0.79 | 0.688  0.134  0.430 | No |
| Burning pain | Hive (Yes) | 0.68 ± 0.31 | 2.18 | 0.029 | Yes |
|  | Age | 0.02 ± 0.01 | 1.90 | 0.057 | No |
|  | Sex (Female) | 0.79 ± 0.65 | 1.23 | 0.219 | No |
|  | Race (White) | -0.08 ± 0.51 | -0.15 | 0.882 | No |
|  | Autonomic diagnosis | -0.18 ± 0.51  (POTS only - NMH only )  0.51 ± 0.87  (POTS and NMH- NMH only )  -0.36 ± 0.54  (Orthostatic Intolerance - NMH only) | -0.35  0.59  -0.66 | 0.727  0.557  0.507 | No |
| Pressure pain | Hive (Yes) | 0.92 ± 0.32 | 2.91 | 0.004 | Yes |
|  | Age | -0.02 ± 0.01 | -1.30 | 0.194 | No |
|  | Sex (Female) | 0.06 ± 0.65 | 0.09 | 0.930 | No |
|  | Race (White) | 0.18 ± 0.51 | 0.35 | 0.726 | No |
|  | Autonomic diagnosis | 0.24 ± 0.53  (POTS only - NMH only )  -0.96 ± 0.88  (POTS and NMH- NMH only )  0.24 ± 0.56  (Orthostatic Intolerance - NMH only) | 0.46  -1.10  0.43 | 0.647  0.272  0.666 | No |
| Electric shock pain | Hive (Yes) | 0.91 ± 0.32 | 2.83 | 0.005 | Yes |
|  | Age | 0.01 ± 0.01 | 1.13 | 0.261 | No |
|  | Sex (Female) | 0.24 ± 0.65 | 0.36 | 0.716 | No |
|  | Race (White) | 0.13 ± 0.51 | 0.25 | 0.805 | No |
|  | Autonomic diagnosis | 0.80 ± 0.56  (POTS only - NMH only )  0.45 ± 0.90  (POTS and NMH- NMH only )  0.49 ± 0.59  (Orthostatic Intolerance - NMH only) | 1.42  0.49  0.83 | 0.156  0.62  0.41 | No |
| Pain provoked by pressure on the painful area | Hive (Yes) | 0.63 ± 0.31 | 2.05 | 0.040 | Yes |
|  | Age | 0.01 ± 0.01 | 0.766 | 0.444 | No |
|  | Sex (Female) | -0.61 ± 0.65 | -0.94 | 0.347 | No |
|  | Race (White) | -0.51 ± 0.51 | -1.01 | 0.313 | No |
|  | Autonomic diagnosis | 0.49 ± 0.52  (POTS only - NMH only )  -0.06 ± 0.88  (POTS and NMH- NMH only )  0.56 ± 0.55  (Orthostatic Intolerance - NMH only) | 0.94  -0.07  1.01 | 0.347  0.946  0.312 | No |
| Provoked by contact with something cold on the painful area | Hive (Yes) | 0.76 ± 0.38 | 2.01 | 0.045 | Yes |
|  | Age | 0.02 ± 0.01 | 1.40 | 0.160 | No |
|  | Sex (Female) | -0.72 ± 0.67 | -1.07 | 0.284 | No |
|  | Race (White) | -0.43 ± 0.53 | -0.81 | 0.419 | No |
|  | Autonomic diagnosis | 0.04 ± 0.63  (POTS only - NMH only )  -0.77 ± 1.21  (POTS and NMH- NMH only )  0.32 ± 0.64  (Orthostatic Intolerance - NMH only) | 0.06  -0.63  0.50 | 0.949  0.527  0.614 | No |
| Tingling | Hive (Yes) | 1.75 ±0.50 | 3.50 | < 0.001 | Yes |
|  | Age | -0.02 ± 0.02 | 1.41 | 0.160 | No |
|  | Sex (Female) | -0.41 ± 1.07 | -0.38 | 0.704 | No |
|  | Race (White) | -0.18 ± 0.78 | -0.23 | 0.818 | No |
|  | Autonomic diagnosis | 0.34 ± 0.70  (POTS only - NMH only )  -0.76 ± 1.05  (POTS and NMH- NMH only )  0.33 ± 0.75  (Orthostatic Intolerance - NMH only) | 0.49  -0.72  0.44 | 0.626  0.469  0.657 | No |
